# Improving *In Vivo* High-Resolution CT Imaging of the Tumour Vasculature in Xenograft Mouse Models through Reduction of Motion and Bone-Streak Artefacts

**DOI:** 10.1371/journal.pone.0128537

**Published:** 2015-06-05

**Authors:** Veerle Kersemans, Pavitra Kannan, John S. Beech, Russell Bates, Benjamin Irving, Stuart Gilchrist, Philip D. Allen, James Thompson, Paul Kinchesh, Christophe Casteleyn, Julia Schnabel, Mike Partridge, Ruth J. Muschel, Sean C. Smart

**Affiliations:** 1 Cancer Research UK and Medical Research Council Oxford Institute for Radiation Oncology, Department of Oncology, University of Oxford, Oxford, United Kingdom; 2 The Institute of Biomedical Engineering, Department of Engineering Science, University of Oxford, Oxford, United Kingdom; 3 Laboratory for Applied Veterinary Morphology, Department of Veterinary Sciences, Faculty of Pharmaceutical, Biomedical and Veterinary Sciences, University of Antwerp, Wilrijk, Belgium; Singapore Immunology Network, SINGAPORE

## Abstract

**Introduction:**

Preclinical in vivo CT is commonly used to visualise vessels at a macroscopic scale. However, it is prone to many artefacts which can degrade the quality of CT images significantly. Although some artefacts can be partially corrected for during image processing, they are best avoided during acquisition. Here, a novel imaging cradle and tumour holder was designed to maximise CT resolution. This approach was used to improve preclinical in vivo imaging of the tumour vasculature.

**Procedures:**

A custom built cradle containing a tumour holder was developed and fix-mounted to the CT system gantry to avoid artefacts arising from scanner vibrations and out-of-field sample positioning. The tumour holder separated the tumour from bones along the axis of rotation of the CT scanner to avoid bone-streaking. It also kept the tumour stationary and insensitive to respiratory motion. System performance was evaluated in terms of tumour immobilisation and reduction of motion and bone artefacts. Pre- and post-contrast CT followed by sequential DCE-MRI of the tumour vasculature in xenograft transplanted mice was performed to confirm vessel patency and demonstrate the multimodal capacity of the new cradle. Vessel characteristics such as diameter, and branching were quantified.

**Results:**

Image artefacts originating from bones and out-of-field sample positioning were avoided whilst those resulting from motions were reduced significantly, thereby maximising the resolution that can be achieved with CT imaging in vivo. Tumour vessels ≥ 77 μm could be resolved and blood flow to the tumour remained functional. The diameter of each tumour vessel was determined and plotted as histograms and vessel branching maps were created. Multimodal imaging using this cradle assembly was preserved and demonstrated.

**Conclusions:**

The presented imaging workflow minimised image artefacts arising from scanner induced vibrations, respiratory motion and radiopaque structures and enabled in vivo CT imaging and quantitative analysis of the tumour vasculature at higher resolution than was possible before. Moreover, it can be applied in a multimodal setting, therefore combining anatomical and dynamic information.

## Introduction

Computed tomography (CT) images are prone to image artefacts which can originate from a range of sources [1, 2]. Some can be partially corrected for by image processing steps but they are best avoided at the acquisition stage. Artefacts introduced by the imaging process can limit the attainable resolution such that the intrinsic resolution of an imaging system cannot be achieved and images are rendered unusable. This often translates into significantly higher x-ray dosing and/or longer scan times than are required to accomplish the best possible resolution in vivo. Image artefacts derived from out-of-field sample positioning, scanner vibration, respiratory motions and strongly absorbing anatomical structures such as bone, can be reduced. Although respiratory gating, for example, has been widely used to address one of these problems, no other strategies have been described to our knowledge to reduce the other confounding factors. While it is important to take care of these artefacts for each study, this report focusses on tumour vasculature imaging in order to improve its visualisation and quantitation in in vivo mouse models of cancer and go beyond imaging the tumour feeding vessels.

While intravital microscopy has played an important role in vascular imaging, spatial coverage is limited and the whole tumour cannot be studied at once [3]. Consequently, a volumetric method is needed and many have resorted to ex vivo vascular corrosion casting. However, the animal must be sacrificed to obtain the image, making longitudinal studies impossible [4]. Therefore, imaging modalities that are non-invasive, volumetric and well-tolerated are of high value. In vivo studies using ultrasound, optical, nuclear, magnetic resonance and CT imaging techniques have been described and were reviewed in this context by Missbach-Guentner et al. in 2011 and more recently by Ehling et al. in 2013 [5, 6]. Unfortunately, many of these present major limitations such as low spatial resolution or low penetration depth preventing visualisation of the tumour vasculature as a whole, especially in mice and rats.

CT is a commonly used in vivo technique to visualise vessels, and has resulted in the visualisation of hepatic blood vessels, large vessels such as the aorta, central veins and arteries, and tumour feeding vessels [7–14]. However, it must be noted that for some applications high contrast agent doses and averaging over multiple scans are required [7, 8]. The latter inevitably results in a high radiation dose, which alters the biology and complicates data interpretation. More recently, Ehling et al were able to visualise vessels using 35 μm3 sized voxels and an iodinated nano-emulsion resulting in in vivo visualisation of large tumour vessels containing up to three branching points [15]. Other attempts to assess tumour vasculature as a whole include vascular density measurements but they are unable to resolve the three-dimensional morphology of tumour blood vessels [11] and read-outs such as diameter, branching factor and branching length cannot be determined.

Given the drawbacks of both vascular casting and vascular density measurements, there still is a gap to be filled for volumetric tumour vascular imaging. CT imaging with its submillimetre resolution is an excellent tool to provide these data. Although it acts on a macroscopic scale, it can not only play a key role as a volumetric method to study 3D tumour vessel architecture over time but also establish links with other vascular read-outs by integrating it in a multi-modal approach. In order to reach its full potential, imaging artefacts resulting from out-of-field sample positioning, vibration, respiratory motion and high density materials such as bone, must be avoided. Therefore, a cantilever imaging cradle loading system, a design used by many manufacturers, is not ideal as this is very sensitive to system and building vibrations. Hence, the purpose of this study is to maximise resolution in in vivo CT imaging of tumour vasculature in tumour bearing mice through minimisation of bone-streak, incomplete projection, scanner vibration and respiratory derived image corruption. Here we describe a new CT imaging set-up that allows longitudinal visualisation of small tumour vessels in mice without the need for complicated image rendering techniques. This was achieved by optimising animal positioning and using a novel imaging cradle. These adjustments were needed to overcome the disadvantages associated with a cantilever-based loading cradle and to avoid motion and bone artefacts. The imaging protocol was optimised and the method was validated against gold standard techniques.

## Materials and Methods

### Ethics statement

All procedures were conducted in accordance with the Animals Scientific Procedures Act of 1986 (UK) (Project License Number 30/2514 and 30/2922 issued by the Home Office). The protocol was approved by the Committee on the Ethics of Animal Experiments of the University of Oxford. All imaging was performed under 1.5–4% isoflurane anaesthesia delivered in room air (4% for induction of anaesthesia, 1.5–2% for maintenance of anaesthesia). Throughout imaging experiments, mice were maintained at 37°C, respiration rate was monitored and all efforts were made to minimize suffering. Mice were housed (n = 5 per cage) in individual ventilated cages in a separate room with 12-h dark and light cycle maintained at 22°C in 50% humidity. All animals were provided with certified rodent diet, filtered water ad libitum, autoclaved bedding material and cage enrichment. No mice were euthanized on welfare grounds. The animals for corrosion casting were culled by an i.v. overdose of pentobarbital to keep the blood vessels intact for casting.

### Animal preparation

The murine adenocarcinoma NT was implanted subcutaneously onto the right flank of 6–7-week-old female CBA/CaCrl mice (n = 26; Charles River). A catheter (PE10, 0.28/0.64 mm internal/external diameter; Linton Instrumentation) was inserted into the lateral tail vein for contrast agent delivery. Exitronnano-12000 (100 μl/20g mouse, i.v.; Miltenyi Biotec) and Omniscan (gadodiamide, 0.5 M, 30 μl; GE Healthcare) were used as contrast agents for CT and Dynamic Contrast Enhanced Magnetic Resonance Imaging (DCE-MRI), respectively. All animals underwent pre- and post-contrast image acquisition. All tubing (anaesthesia delivery and respiration balloon) and metal (heating blanket) was kept outside Field-Of-View (FOV) of the CT (Inveon PET/CT, Siemens Healthcare).

### Cradle design

**Fig 1:**
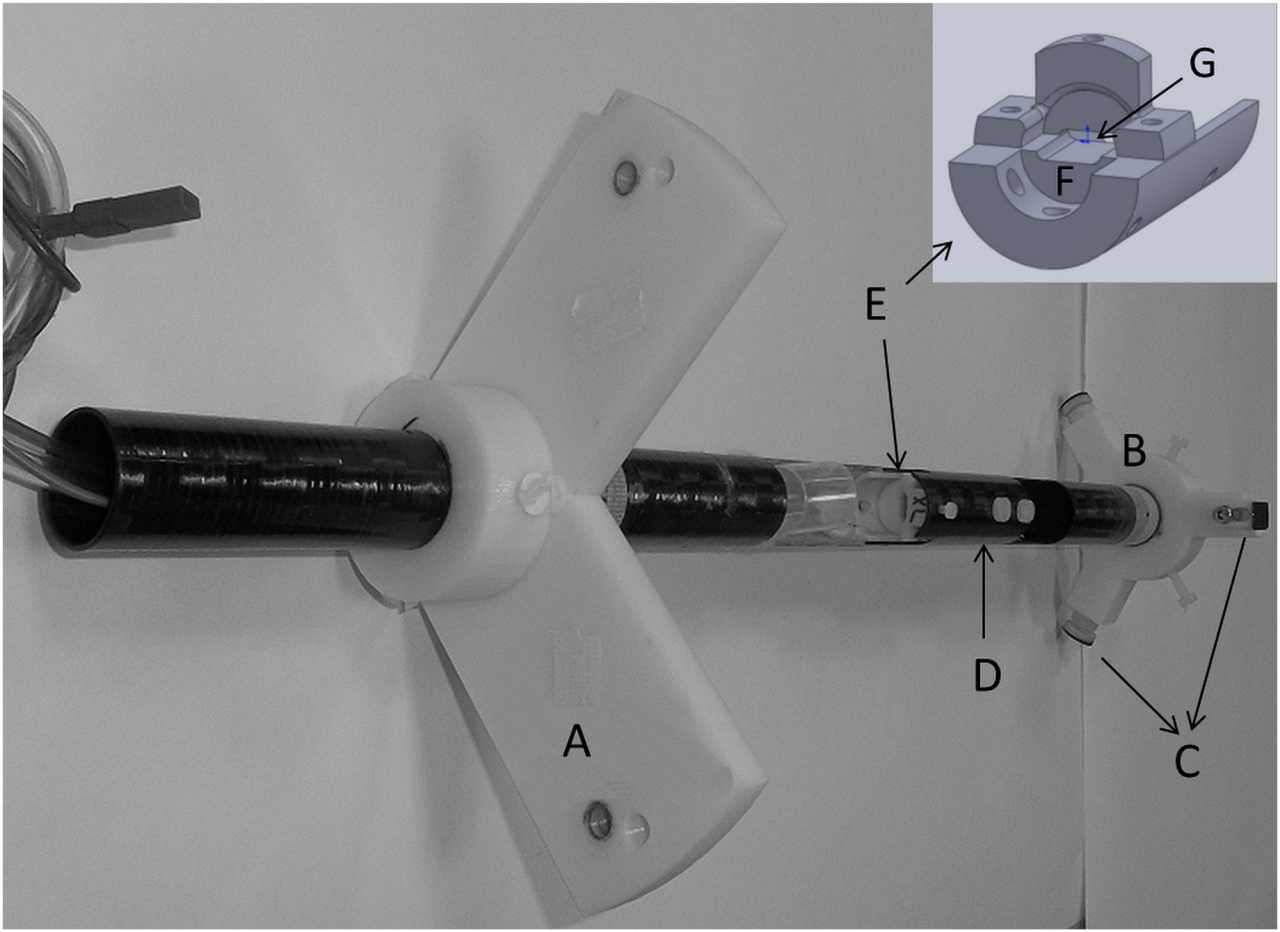
Diagram of the custom cradle. The different components that make up the custom cradle are A: front mounting ring, B: back mounting ring, C: Adjustable feet, D: cradle ‘roof’, E: tumour holder, F: platform to support the tumour, G: slit to allow tumour positioning.

The standard support cradle as supplied by the manufacturer consists of a carbon fibre imaging bed (25 mm width for high-resolution CT imaging), which is mounted on a cantilever system for translation into the imaging FOV. This cantilever set-up is sensitive to motion arising from gantry rotation and room/machine vibrations as its fulcrum defines the first degree of freedom for vibrations to propagate into uncontrolled motion. In addition, up to half of the FOV is used when optimal bed positioning is applied for the supplied cradles, making it difficult to position the animal within the boundaries of the FOV at high system magnification setting. To avoid the cantilever system and to maximize the use of the FOV, a custom built cradle containing a tumour holder was developed (Fig 1). It consists of a carbon fibre tube (24/27 mm internal/external diameter; Carbon Fibre Tubes Ltd), which can be rigidly fixed into the CT gantry so both the cradle and imaging system are vibrating in sync. Two removable mounting rings provide the support for the cradle and make sure that the cradle can be centred into the CT-FOV. The front mounting-ring is secured to the gantry using the bore tunnel screw holes. The back mounting-ring is fixed into the PET bore using adjustable feet coated with rubber to prevent it from slipping. A plastic tumour holder was designed to isolate the tumour from the rest of the body without harming the animal or clamping of the blood flow to the tumour. The base of the tumour holder is permanently fixed into the cradle, broadened to provide rigidity and contains a platform for the tumour to rest on. The top half of the tumour holder is fixed to the bottom half using two plastic screws. A slit between the top and bottom half of the holder allows positioning of the tumour. The slit size can be adjusted to optimally fix tumours of different sizes ranging from approximately 25–400 mm^3^. Additional rigidity was provided by placing a carbon fibre ‘roof’ on top of the tumour holder at the tumour end to complete the cradle. The cradle mounting rings and tumour holder were designed in Solidworks-2014 and printed using a HP Designjet 3D Printer.

### Vascular corrosion casting

The procedure previously described by Trachet et al [16] was performed. In short, mice were sacrificed by an overdose of pentobarbital i.v. The left heart ventricle was exposed and catheterized with a 25 gauge catheter (Venoflux). Opening the thoracic cage was sufficient to allow for the outflow of excessive blood. A total volume of 2–3 ml casting polymer (Batson's #17 Plastic Replica and Corrosion Kit; Polysciences; 5 ml base monomer, 0.75 ml catalyst, pinch of red pigment, 2 drops of promoter) was injected free-hand. The mouse bodies were immersed in cold water during the exothermic polymerization reaction. Maceration took place at 22°C for 24 hours in a 25% KOH (VWR) in water solution. Then, the casts were rinsed gently with tepid streaming tap water and transferred to a 10% HCl (Sigma-Aldrich) solution in water for 1 hour to dissolve the bones. Again, casts were rinsed gently with tepid streaming tap water. Finally, they were transferred onto a filter paper, dried at 22°C and evaluated by CT using the protocol described above. The corrosion casts were imaged using the custom cradle without the tumour holder in place. The vascular corrosion casts were used to check for artefact-related structures in the CT vessel renderings by visually comparing the CT images to the actual vascular replicas.

### Image acquisition protocols

#### CT imaging

For in vivo imaging, the scanner was operated using the following standard high-resolution settings: X-ray tube settings of 65 kV and 500 μA (nominal tungsten anode spot size of 46 μm) without additional tube filtration, a 300 ms exposure time per projection, 540 projections, a 360° continuous rotation, a high system magnification (source-to-centre = 370.6 mm; source-to-detector = 102.2 mm), a binning factor of 4, a matrix size of 3072 x 2048 and gating was disabled. Furthermore, a resting time of 25 min was applied following animal loading and cradle positioning before imaging was started to allow the system to come to mechanical equilibrium.

Vascular corrosion casts were imaged using the following standard high-resolution settings: X-ray tube settings of 65 kV and 190 μA (nominal tungsten anode spot size of 15 μm) without additional tube filtration, a 3500 ms exposure time per projection, 720 projections, a 360° stepwise rotation using a settling time of 5 s, a high system magnification, a binning factor of 2 and a matrix size of 3072 x 2048.

All CT images were reconstructed using the standard Feldkamp algorithm for cone beam CT reconstruction, with a Shepp-Logan filter with cut-off at the system Nyquist frequency, resulting in an image matrix of 768 × 768 × 512 with an isotropic voxel size of 37.2 μm for the in vivo protocol and 1536 × 1536 × 1024 with an isotropic voxel size of 18.6 μm for the vascular corrosion cast protocol.

#### DCE imaging

DCE-MRI was performed at 4.7 T (Varian, VNMRS) using a RF and gradient-spoiled 3D gradient echo sequence (TE 0.6, TR 1.15, α = 5 nominal, image matrix 128 x 64 x 64, 422 μm isotropic resolution) and a home-built surface RF-coil. Five hundred images were acquired over 10 minutes. Gadodiamide was automatically infused over 5 s using a syringe pump (Harvard Apparatus) into the lateral tail vein, triggered at the start of scan 51/500.

### Evaluation of spatial resolution

The maximum achievable spatial resolution of the CT scanner was determined by using a mouse-sized phantom containing different nylon filament sizes (50, 100, 210, and 370 μm in diameter) and calculating the full-width at half-maximum (FWHM) from the line profiles of the filaments. Phantoms were scanned using the abovementioned protocols at binning factors of 2 and 4, and at medium and high magnifications. For each factor and magnification, measurements were obtained from 10 line profiles using Matlab (Mathworks) and converted to micrometers using the isotropic voxel size for that scan.

### Evaluation of cradle immobilisation

Motion originating from the cradle itself following mounting into the CT gantry was evaluated by acquiring 10 consecutive images of the empty cradle. Motion was assessed by subtracting panes of sequentially acquired images in ImageJ [17]. This procedure was repeated 10 times. To illustrate the motion-related artefacts associated with the standard support cradle as supplied by the manufacturer which is mounted on a cantilever system, a 5 ml syringe containing tap water was imaged using the original cradle set-up.

### Evaluation of the tumour holder

#### Bone-streaking

A phantom containing mouse bones in 1% agarose in water (w/v) (Sigma-Aldrich) was imaged using the custom cradle and the in vivo CT acquisition parameters. The phantom was placed perpendicular or parallel to the axis of rotation of the CT scanner as shown in S1A Fig. The parallel orientation mimics the in vivo set-up where the tumour holder separates the tumour from the rest of the body. Subsequently, bone-streaking was evaluated in vivo (n = 26). Both phantom and in vivo data were assessed for the presence/absence of bone-streaking artefacts.

#### Tumour immobilisation and tumour blood flow

Tumour immobilisation was evaluated by acquiring fluoroscopy and pre- and post-contrast CT imaging, whilst preservation of tumour blood flow was assessed by DCE-MRI. The same animals (n = 5) were used for both examinations. Fluoroscopy, CT and DCE-MRI imaging was performed sequentially during the same anaesthetic session using the same cradle.

Fluoroscopy, acquiring an image every second, was performed during the settling time of the cradle using the in vivo protocol settings as described above. Images displaying the standard deviation of mean intensity were created in ImageJ. Subsequently, pre- and post-contrast CT imaging was performed and subtraction of the sequentially acquired images was carried out in ImageJ.

DCE-MRI was performed as described above. Pre-contrast enhanced images were subtracted from post-contrast images at 10 minutes p.i. to reveal gadolinium-avid tumour regions. The subtraction images were created in ImageJ.

### Vessel image rendering and quantitation

ImageJ, Image Registration Toolkit (IRTK) and Matlab were used for image processing. First, the pre- and post-contrast images were normalised based on their intensity histograms before being co-registered using a non-rigid b-spline grid registration [18, 19]. Image registrations were performed in order to minimise the impact of small, residual tissue motion. Next, the pre-contrast image was subtracted from its corresponding post-contrast image and the 3D vessel structures were segmented using intensity thresholding. Furthermore, a multi-scale vesselness filter was used to enhance vessel structures and improve small vessel delineation [20]. This vesselness map was then thresholded to produce a vesselness map segmentation. Vessel quantitation was performed by creating branching maps of the tumour vasculature based on a skeletonization of the vesselness map segmentations. The diameter of segmented vessels was calculated using a distance map approach.

### Dosimetry

The dose per CT scan and the associated dose distributions were determined using EBT3 gafchromic film (Vertec Scientific Ltd) as described previously [21]. Briefly, one film was sandwiched between two identical semi-cylinders of WT1 water equivalent material, which made up a cylindrical mouse phantom. Another film was placed on top of the phantom to determine the ‘skin dose’. The exposed film strips were processed, optical density values were corrected and converted to dose values and the dose per CT scan was determined.

## Results

### Evaluation of spatial resolution

FWHM of different sized filaments was measured to determine the spatial resolution of the CT scanner. Spatial resolution reached a maximum of 76.7 ± 8.5 μm when a binning factor of 2 and the highest magnification were used (Table 1).

**Table 1:** Full-width at half-maximum (FWHM) measured at two different binning factors and magnifications using nylon filaments of four sizes.

Wire diameter (μm)	FWHM (μm)			
	Binning factor 4		Binning factor 2	
	Medium magnification	High magnification	Medium magnification	High magnification
**370**	380.1±19.9	385.9±5.1	387.7±15.0	395.4±5.4
**210**	294.0±28.6	228.1±6.8	225.8±15.5	238.4±7.0
**100**	251.8±14.4	171.5±13.2	146.3±6.4	130.7±8.1
**50**	250.8±49.4	146.9±11.0	126.6±14.1	76.7±8.5

Data represent mean ± SD from 10 line profiles.

### Evaluation of cradle immobilisation

The new cradle fitted within the CT imaging FOV as shown in S2A Fig. The cradle made full use of the available FOV and larger samples as compared to the manufacturer’s cradle assembly could be positioned within the same FOV. Besides positioning the sample in the tumour holder and fixing the cradle in place, no extra care is needed to centre the object of interest within the FOV. As a result, out-of-field artefacts arising from incomplete projections are inherently avoided. The forces resulting from fixing the cradle into the CT gantry, or transverse loading could have caused the cradle to bend and deflect from its original position. However, repeated imaging showed that these deformations settle over time as the subtraction images become featureless after the 2^nd^ to 3^rd^ scan post fixing (S2 Fig). As a result, a minimum settling time of 4 scans, or 24 minutes, was taken into account to avoid any motion arising from the imaging cradle itself. The motion-related artefacts associated with the standard support cradle as supplied by the manufacturer are illustrated by S3 Fig and manifested themselves as shading in the reconstructed images and a misregistration of up to 9 pixels (6.3 ± 1.0 pixels; n = 5) for two consecutive image acquisitions.

### Evaluation of the tumour holder

#### Bone-streaking

The phantom study indicated that serious streaking artefacts occur when the sample is placed perpendicular to the axis of rotation of the CT scanner (S1B–S1D Fig). In contrast, bone-streak artefacts were avoided in the region of interest when the sample was placed parallel to the axis of rotation (S1E–S1G Fig). In vivo imaging using the custom cradle and the tumour holder applies the latter orientation. Indeed, the tumour holder separates the tumour from the rest of the mouse body and in effect removes the bones from the imaging direction. As a result, no bone-streaking artefacts were observed in the tumour region when the tumour holder was applied. A representative image is shown in S1H Fig.

#### Tumour immobilisation and tumour blood flow

The custom cradle provided a rigid backbone to fix the tumour holder in place. Fluoroscopy was used to evaluate both cradle and tumour immobilisation. This test not only confirmed that the cradle deformations settle over time, but also showed that the tumour holder is capable of keeping the tumour stationary whilst the remainder of the body is still subject to respiratory motion. The latter was apparent by studying the standard deviation of the mean images: the tumour area was featureless whilst the rest of the body still appears (S4 Fig).

**Fig 2:**
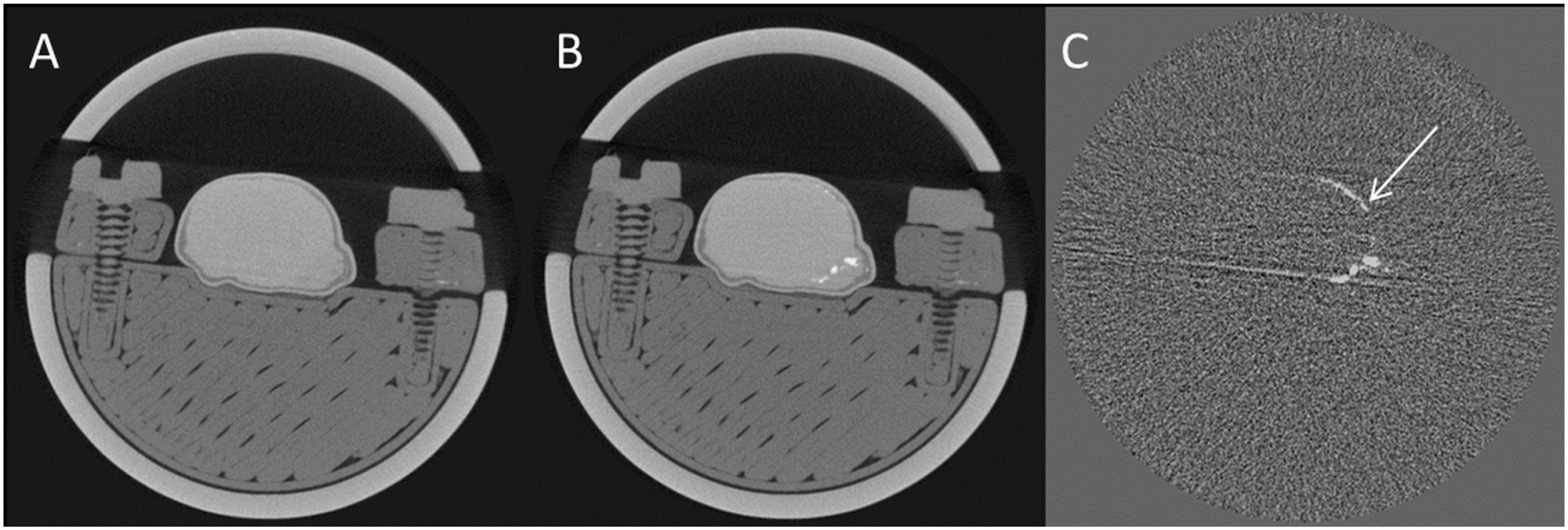
CT imaging using the proposed method. A centre slice through the tumour is shown. A: pre-contrast CT, B: post-contrast CT, C: subtraction image (post—pre). The white arrow indicates a 2-pixel discrepancy between the pre- and post-contrast image.

CT imaging was performed following a 24-minute settling time. Subtracting the pre-contrast from the post-contrast image again confirmed the absence of cradle, tumour holder and bulk tumour motion (Fig 2). As a result, the subtraction images only showed the presence of contrast agent. Apart from the five mice reported above, a total of 26 paired CT images were evaluated for tumour immobilisation. Although the bulk of the tumour was stationary, part of the tumour rim showed a low level deformation: an average maximum discrepancy of 2.0 ± 0.9 pixels (n = 26) was observed between the pre- and post-contrast image.

**Fig 3:**
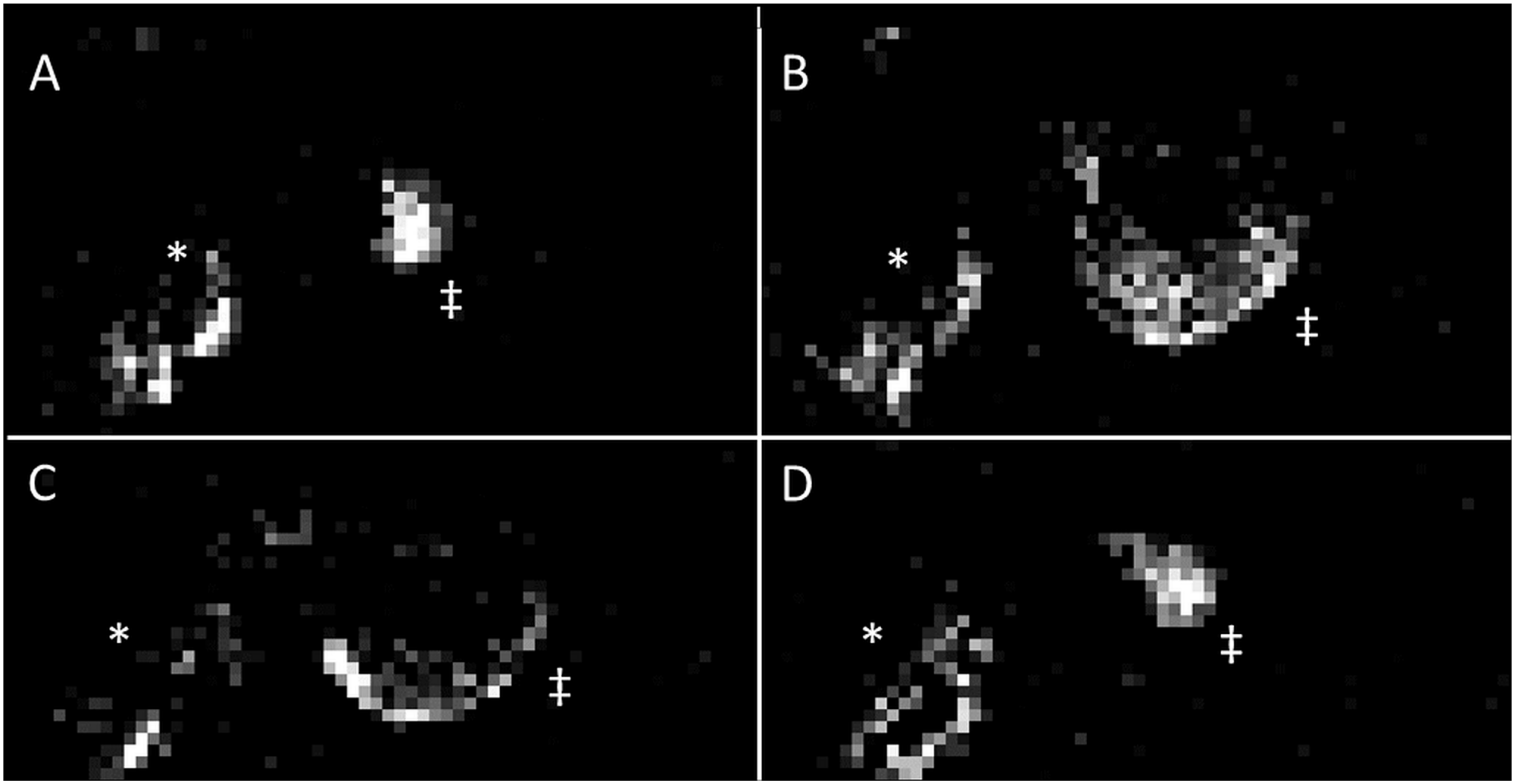
Evaluation of tumour blood flow following tumour immobilisation. DCE-MRI was performed sequentially to CT in the same mice during the same anaesthetic session and using the same cradle. Pre-contrast enhanced images were subtracted from post-contrast images at 10 minutes p.i. to reveal gadolinium-avid tumour regions. A-D: The gadolinium uptake pattern for each 8th slice of the tumour is depicted. Results for the same mouse as featured in Fig 2 are shown. The animal’s body is indicated by a *, its tumour by a ‡.

**Fig 4:**
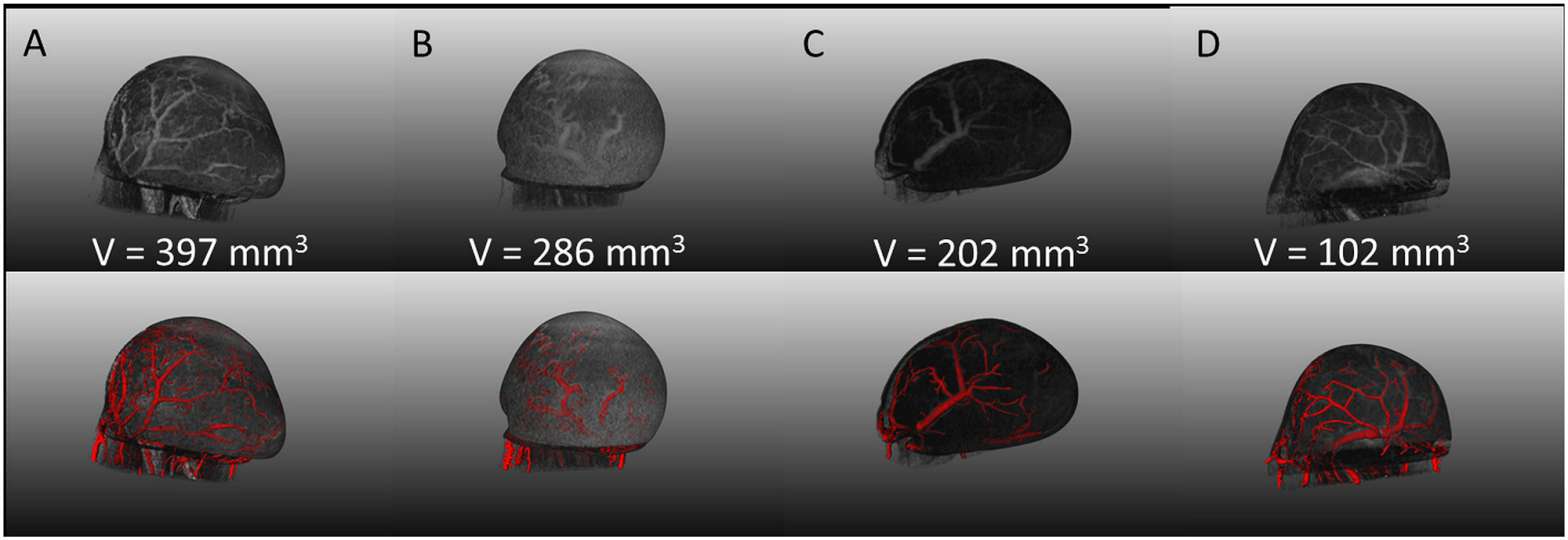
Tumour vessel image rendering. Column A-D: A showcase of four representative mice, displaying a range in tumour volumes, is presented. 3D vessel segmentations based on intensity thresholding are portrayed in the top row and are overlaid with the tumour surface. The bottom row presents a composite image of the vessel segmentations with the vesselness map segmentation. Tumour ‘C’ portrays the same animal as shown in Fig 5.

Although vessel contrast enhancement was clearly observed following CT contrast agent injection, DCE-MRI was performed in the same mice in a multi-modal setup (Fig 3). A typical heterogeneous uptake pattern emerged: regions of high blood flow existed at the rim of the tumour and normal muscle tissue whilst the tumour centre was less perfused. These results are in line with the anatomical CT images that showed a higher density of vessels at the rim of the tumour as compared to the centre (Fig 4). Moreover, this confirmed that tumour blood flow was preserved during tumour immobilisation. A representative DCE-MRI image is presented in Fig 3.

### Vascular corrosion casting

**Fig 5:**
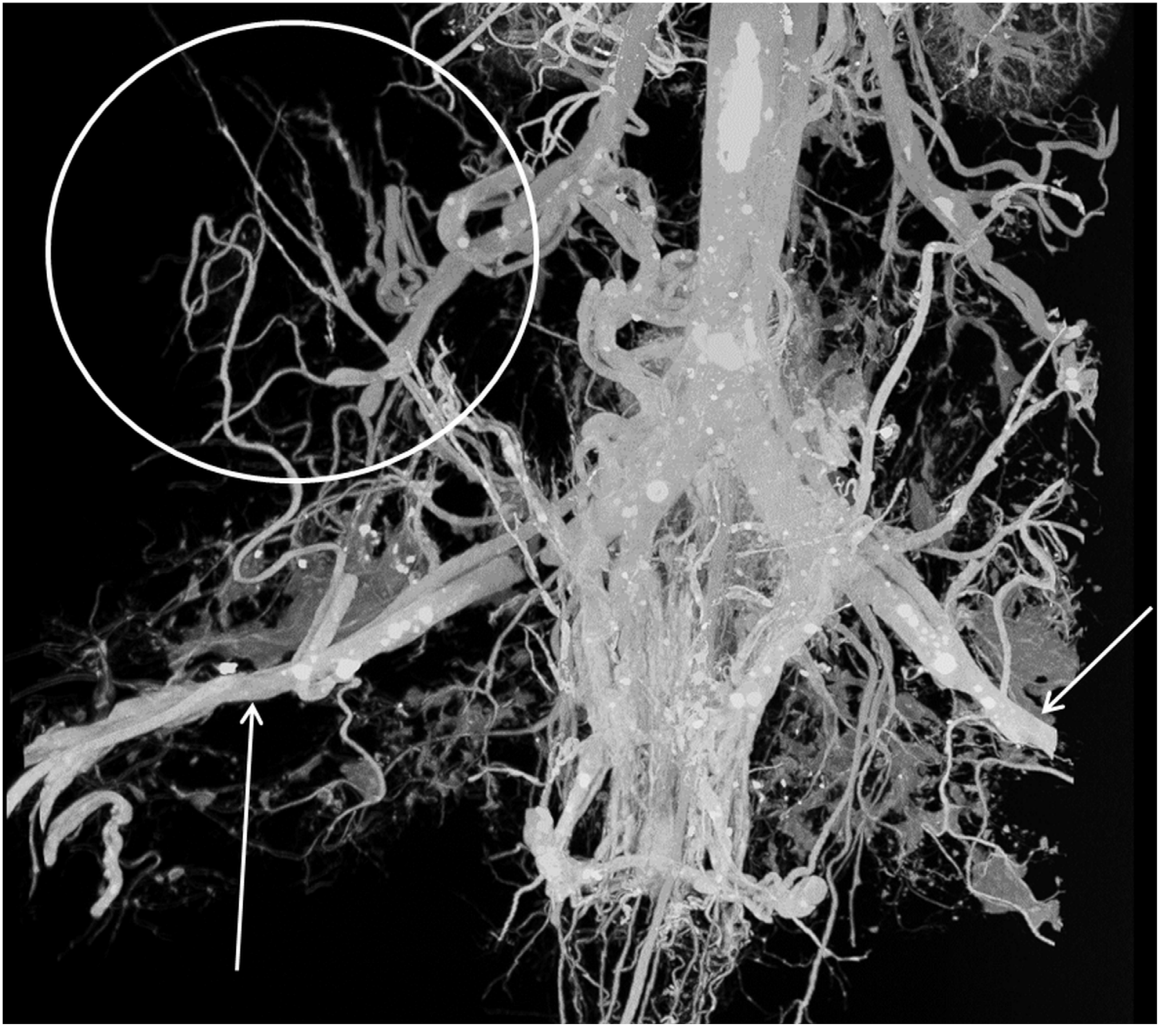
3D maximum-intensity-projection vessel rendering of a vascular cast for the same animal as Fig 4C. The box indicates tumour position whilst the arrows indicate the a. femoralis.

Anatomical dissection of mouse cadavers showed that the major feeding vessels of the xenograft tumours originated from the arteria epigastrica cranialis and caudalis. Therefore, vascular corrosion casts were obtained by perfusing the casting solution through the left heart ventricle so that both arteries could be targeted. Visual inspection showed that for all animals the major tumour vessels could be cast. Although the plastic vascular replicas were less radiopaque as compared to the CT contrast agent they could still be visualised by adjusting the CT acquisition parameters. A typical 3D maximum-intensity projection rendering of a vascular cast is presented in Fig 5. Since the vascular replicas show nothing but the vascular network, the CT image renderings of the same cast can be easily compared to cast itself and visually inspected for the presence of extra, artefact-related, features. In our case, only vessels and no additional structures, such as vessel duplicates, were observed in the CT images.

### Vessel image rendering and quantitation

**Fig 6:**
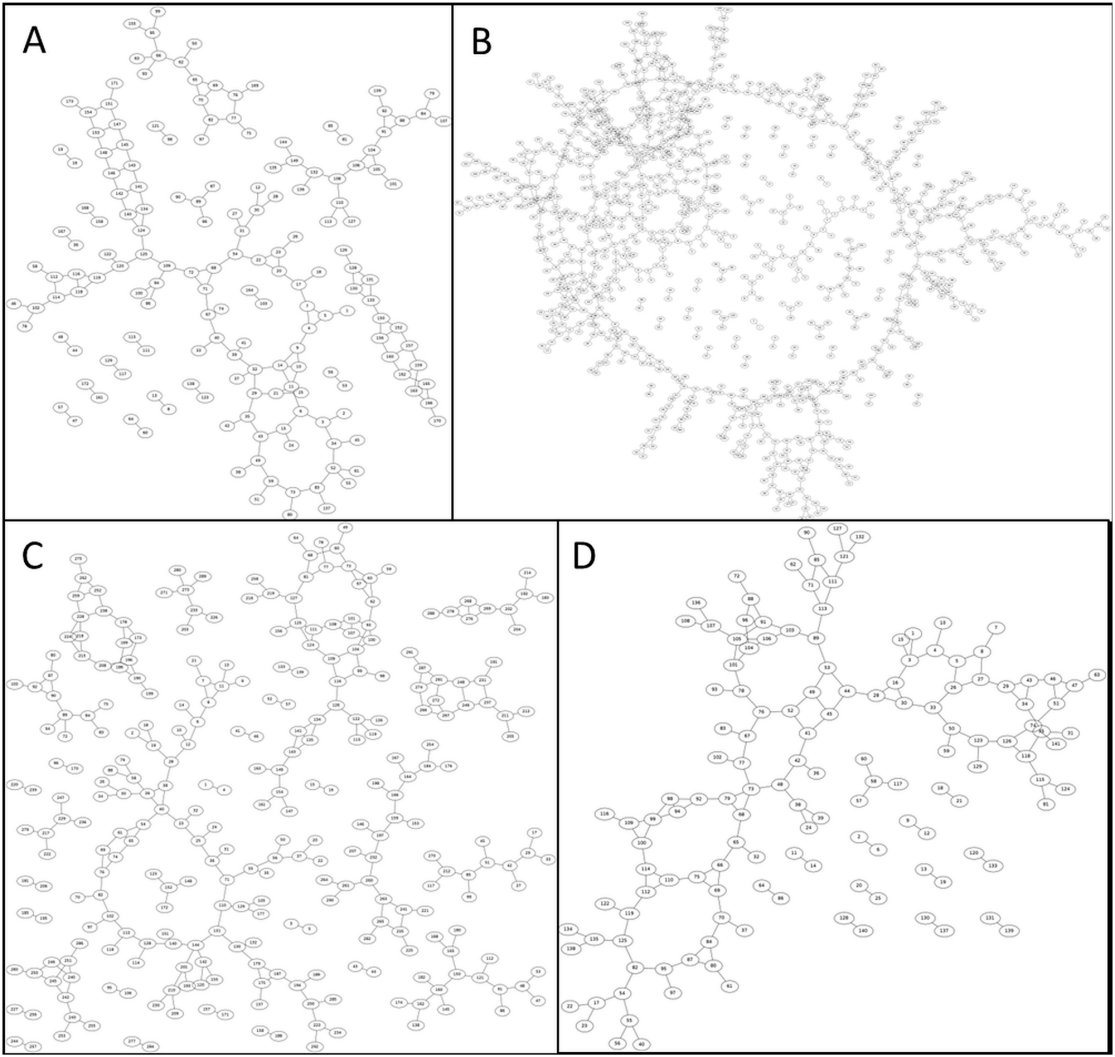
Branching maps of the tumour vasculature. These are based on a skeletonization of the vesselness map segmentations and illustrate the degree of branching and the number of branches for each tumour. A-D: A showcase of branching maps for four representative mice, displaying a range in tumour volumes, is presented. The maps correspond to the same tumours as portrayed in Fig 4. The same annotations were used to identify the tumours; i.e. Fig 4 column A corresponds to Fig 6A.

**Fig 7:**
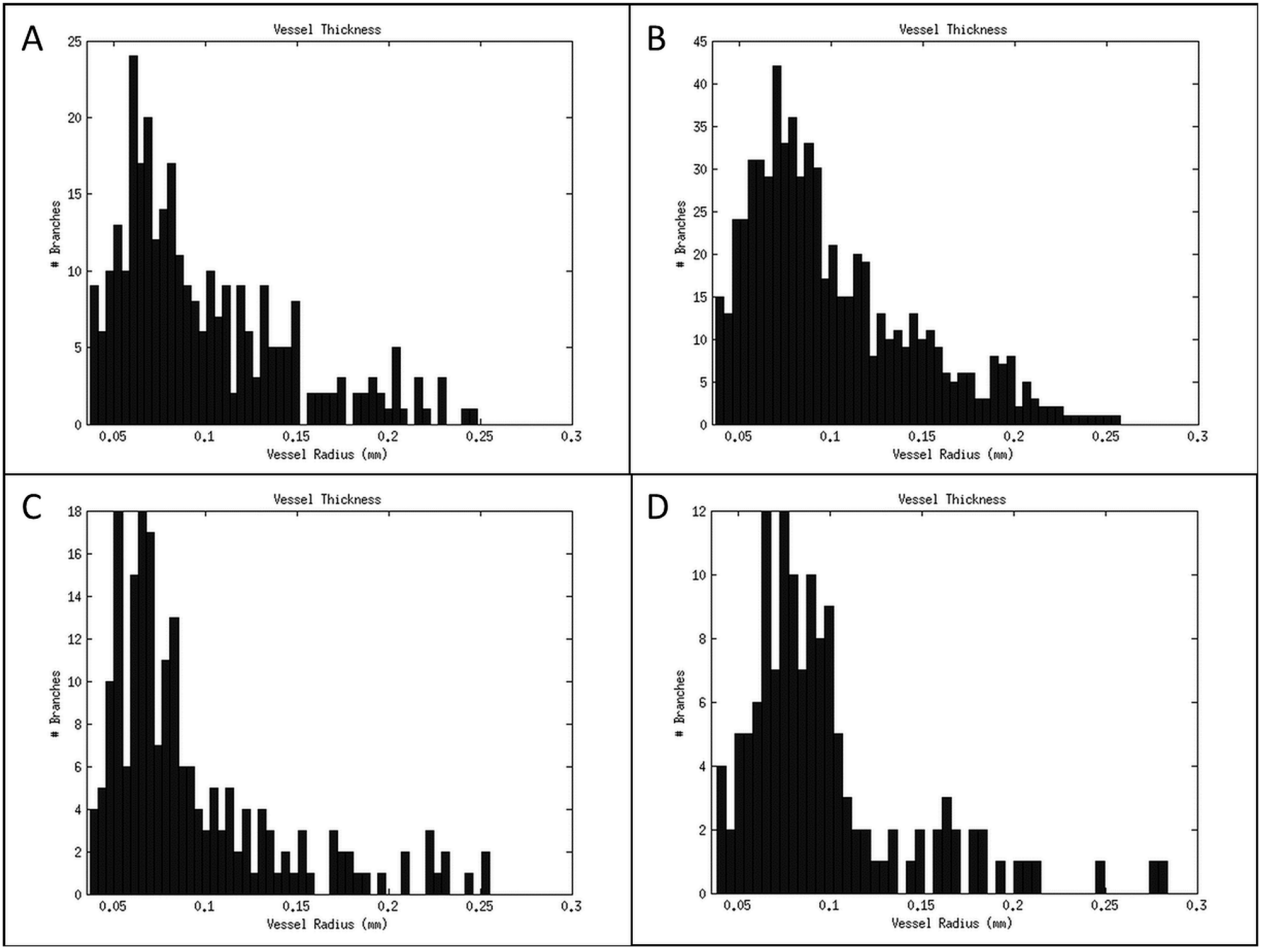
Analysis of tumour vessel diameter. The diameter of each tumour vessel was determined using a distance map approach and plotted as a histogram. A-D: Vessel diameter histograms for four representative mice, displaying a range in tumour volumes, are presented. The histograms correspond to the same tumours as portrayed in Fig 4. The same annotations were used to identify the tumours; i.e. Fig 4 column A corresponds to Fig 7A.

Bone and out-of-field streaking were eliminated and physiological and scanner derived motion artefacts were reduced by almost 80%. As a result, the tumour vessels could be imaged with live-animal CT with a nominal 77 μm resolution by subtracting pre-and post-contrast acquisitions. The remaining low level deformation that was observed in some of the animals could be adjusted by applying a straightforward non-rigid b-spline grid registration of the pre-and post-contrast images. A showcase of 3D intensity thresholded vessel renderings for multiple tumours of different sizes (approximate volumes of 100, 200, 300 and 400 mm3) is presented in Fig 4, together with their respective composite image containing the vessel segmentation and vesselness map segmentations. Vessel diameter could be extracted from the former image whilst the latter can be reduced to its skeletal structure from which the degree of branching and the number of branches can be determined as illustrated by Fig 6 and Fig 7, respectively.

### Dosimetry

Dosimetry was performed to establish the radiation dose to the animal for each imaging session as dose accumulation is an important experimental parameter in longitudinal studies. The weighted average animal dose and the weighted average skin dose were 1.7 Gy and 6.1 Gy, respectively.

## Discussion

Non-invasive longitudinal imaging of the tumour vascular network and subsequent vessel segmentation is not a trivial task. Thus far, only larger vessels such as the tumour feeding vessels could be segmented, visualised and analysed and this is testament to the many hurdles that need to be overcome [7, 8, 12, 13, 15, 22]. One of these is the choice of the contrast agent and although this is not the focus of this paper, it is an integral part of the CT imaging procedure. As shown by others, contrast resolution differs widely amongst contrast agents and the latest generation of blood-pool contrast agents, of which Exitronnano-12000 is an example, outperform the classical water soluble ones whilst using a fraction of the dose [7, 8, 10, 13, 23]. For these reasons, our studies have been performed using Exitronnano-12000.

More importantly, however, is the avoidance of imaging acquisition artefacts, since these are detrimental to spatial resolution and 3D vessel segmentation. Although previous papers already report on high resolution CT imaging of tumour vessels, so far only the largest tumour vessels could be segmented showing few branching points [7, 8, 11–13, 15, 22, 24]. One of the reasons for this low amount of image detail might be the existence of artefacts that are best avoided at acquisition stage as they are difficult to remove during image processing. Indeed, motion artefacts can blur, replicate or mimic vessel structures whilst streak artefacts emanating from radiopaque structures such as bones and tubing can mimic vessel-like structures [1, 2]. Consequently, all will have a dramatic effect on the study outcome as they create either false positive or false negative results. Unfortunately, most preclinical CT systems use a cantilever system to position the imaging cradle into the imaging FOV. This design is sensitive to vibrations, not only emanating from the environment but also from gantry rotation. To circumvent this problem, a custom cradle was designed that could be fixed within the gantry itself. In this way, both the cradle and imaging system are rigidly attached in the same frame, thus vibrating in sync, and images appear free of motion artefacts as was illustrated by S2 Fig. The flexibility of the custom cradle design will also allow adjusting it to fit other imaging systems than used in this study. Lastly, once the cradle is fixed in the imaging system, one does not have to worry about sample positioning as it will automatically fit within the FOV and avoid incomplete projection artefacts.

Another major improvement was the introduction of the tumour holder which distanced the tumour from the bones so bone artefacts were avoided. Fortuitously, this holder also served another purpose as it kept the tumour stationary to within 200 μm so gating became superfluous. Moreover, circumventing gating allows for faster imaging and makes the imaging procedure more operator-friendly since gating control signals do not need to be monitored. Although the exposure time per projection can be set by the scanner, it is not limited anymore to the inter-breath interval and fluctuations in respiration rate will not re-introduce motion artefacts. Thus, a higher instantaneous x-ray dose can be applied, accelerating the imaging process without reducing the signal-to-noise ratio. Although the tumour holder worked well, as illustrated by S4 Fig, long term drift, rather than respiratory motion artefacts, still caused the tumour to move a few pixels over the duration of the acquisition. But, image fidelity, which was maintained by avoiding respiration motion artefacts, enabled the use of pre-and post-contrast image co-registration to correct for this minimal offset. Consequently, the tumour vessels could be rendered reproducibly in vivo by using a simple maximum-intensity-projection and this to a level that could visualise the architectural structure beyond just the feeding vessels with smaller vessels clearly resolved as the imaging system could be operated at its maximum achievable resolution. Previous attempts at vessel imaging by CT in live mice include the use of high contrast agent doses, an isolator in combination with high contrast agent doses and image averaging, and customised contrast agents [7, 8, 15, 22, 23]. Others used second generation contrast agents such as Isovist-300 and eXia160 and fast image acquisition protocols but did not apply respiratory gating to avoid motion artefacts [11–13, 24]. However, these attempts were only able to resolve the largest vessels, such as central arteries and veins and their bifurcations towards the tumour. The tumour vessels themselves were not resolved clearly and appeared as a haze. The same is still true for contrast enhanced MR angiography [25]. Although we still are only able to image at a macroscopic scale, as so well described by Cebulla et al [3], the amount of detail that can be observed in vivo at a nominal resolution of 77 μm a resolution similar to that obtained ex vivo in previous reports, when using the proposed method has increased [7, 8, 12, 13, 15, 22, 24]. Indeed, imaging beyond the feeding vessels has become feasible as many vessel junctions, both at the tumour rim and centre, can be visualised.

Furthermore, DCE-MRI and CT were performed sequentially in the same mice, during the same imaging session, using the same cradle and tumour holder to study the blood flow to the tumour. DCE-MRI was chosen as it is common in oncology to provide functional information about the tumour vasculature with results reflecting a composite of tumour perfusion and vessel permeability [26]. Gadolinium uptake kinetics in muscular tissue was as fast as in well-perfused areas of the tumour, i.e. tumour rim. This not only indicated that the blood flow to the tumour was functional but also that this approach is still amenable to multimodal research.

Next, the images obtained by in vivo CT imaging were compared to CT images of vascular corrosion casts. Although specific artefacts were minimised by using the set-up as was shown in S4 Fig and Fig 4, residual imaging artefacts and inhomogeneities have the potential to mimic vessel-like structures, especially when tubeness filters, such as Frangi-based algorithms, are used for vessel segmentations. Artefacts would, however, show up as highly ordered structures (e.g. rings, striations and replicated vessels) or added noise upon visual inspection of the segmented vessel images. This was not observed and, together with the analogous structures seen when comparing the imaging of vascular casts to the replicas themselves, strongly indicates that we are indeed visualising vessels and not artefacts. Consequently, the rendered images can be quantified and vessel characteristics such as diameter, volume, branching can be quantified and analysed.

Lastly, dosimetry was taken into account, as this imaging set-up will ultimately be used in longitudinal studies. Unfortunately, the Inveon PET/CT system delivered an average whole body dose of 1.7 Gy per acquisition resulting in 3.4 Gy for the reported imaging workflow which is equivalent to those using long scan times and image averaging [8]. While this dose is significant, causing biological damage [21], this can be remedied by switching to the latest generation CT system with higher sensitivity detectors. Such systems are much faster and the latest developments have resulted in a significant reduction in absorbed dose whilst maintaining high quality image output [27, 28]. Based on the required imaging parameters, we estimate that the use of high sensitivity detectors would result in a 4- to 20-fold reduction in radiation dose for our workflow. However, similar modifications to the imaging cradle and use of the tumour holder would still be required.

## Conclusion

The presented imaging workflow using a novel imaging cradle and tumour holder minimised image artefacts arising from scanner induced vibrations, respiratory motion and radiopaque structures such as bone. The proposed method provides a general solution for motion and radiopaque related artefacts for when they occur so that improved image quality and highly detailed images can be extracted using the same acquisition protocol. It allows imaging of the tumour vascular network at 77 μm resolution providing anatomical and dynamic information within the same animal. Moreover, quantitative measurements such as vessel diameter and branching can be derived from the images. Applying this method will aid biology studies of the tumour microenvironment and enhance the implementation of the 3Rs (replacement, reduction and refinement). The only limitation left is the radiation dose, but this can be solved by transferring this same system to the latest generation of high-resolution CT scanners.

## Supporting Information

S1 Fig Evaluation of bone streaking artefacts. A: a diagram explaining the positioning of the phantom in the cradle; B-D: Phantom placed parallel to the imaging direction which resulted in the presence of bone-streak artefacts throughout the region of interest; C: Positive and negative streaking is observed; D: Negative streaking attributed to the bones could be observed; E-G: Phantom placed perpendicular to the imaging direction, similar to the in vivo situation using the tumour holder, which resulted in the absence (G) of bone-streak artefacts throughout the region of interest; H: In vivo imaging using the tumour holder to separate the bones from the tumour. Bone-streak artefacts were avoided in the region of interest, the tumour.
(TIF)

S2 Fig Evaluation of cradle immobilisation. Repeated imaging of the empty custom cradle over time illustrating the level of deformation following mounting into the imaging system. Each CT image took approximately 7 min and subsequent images were acquired without delay. The first scan was started immediately following mounting of the cradle into the imaging system. The same slice is shown for each image. A: CT image; B-J: Subtraction of two subsequent images with B: image 2–1; C: image 3–2; D: image 4–3; E: image 5–4; F: image 6–5; G: image 7–6; H: image 8–7; I: image 9–8; J: image 10–9. The subtraction images become featureless after the 2nd to 3rd scan post fixing.
(TIF)

S3 Fig Evaluation of cradle immobilisation. A water phantom was used to assess the sensitivity of the manufacturer’s cantilever system to machine vibrations. Motion artefacts appeared as shading or streaking in the reconstructed CT image as indicated by the arrows.
(TIF)

S4 Fig Evaluation of tumour immobilisation using the tumour holder. Fluoroscopy was performed during settling time of the custom cradle. Imaging was initiated immediately after mounting the cradle into the imaging system. Standard deviation of the mean is shown for 50 subsequent images. The bright edges show areas of high standard deviation and correspond to areas of large motion. A: Fluoroscopy image, B: 0–2 min, C: 5–7 min, D: 15–17 min, E: 22–24 min. The remaining wave patterns result from a temporal stability of the X-ray source.
(TIF)
