# Supplementary figures and images for: Improving In Vivo High-Resolution CT Imaging of the Tumour Vasculature in Xenograft Mouse Models through Reduction of Motion and Bone-Streak Artefacts
Source: PLoS One. 2015 Jun 5;10(6):e0128537. doi: 10.1371/journal.pone.0128537 (PMC4457787; doi:10.1371/journal.pone.0128537)

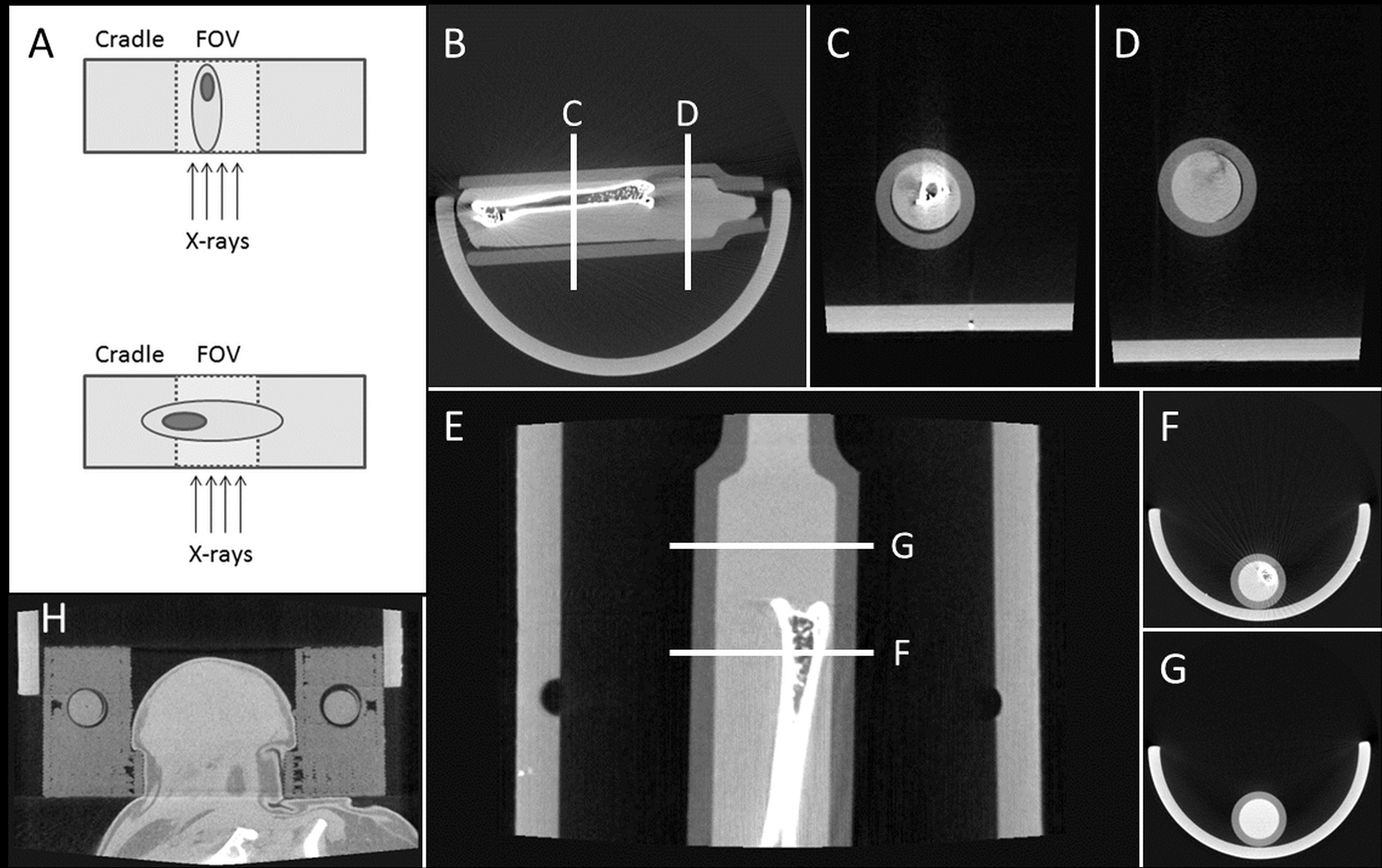

Supplement: S1 Fig — A: a diagram explaining the positioning of the phantom in the cradle; B-D: Phantom placed parallel to the imaging direction which resulted in the presence of bone-streak artefacts throughout the region of interest; C: Positive and negative streaking is observed; D: Negative streaking attributed to the bones could be observed; E-G: Phantom placed perpendicular to the imaging direction, similar to the in vivo situation using the tumour holder, which resulted in the absence (G) of bone-streak artefacts throughout the region of interest; H: In vivo imaging using the tumour holder to separate the bones from the tumour. Bone-streak artefacts were avoided in the region of interest, the tumour. (TIF) [file pone.0128537.s001.tif]

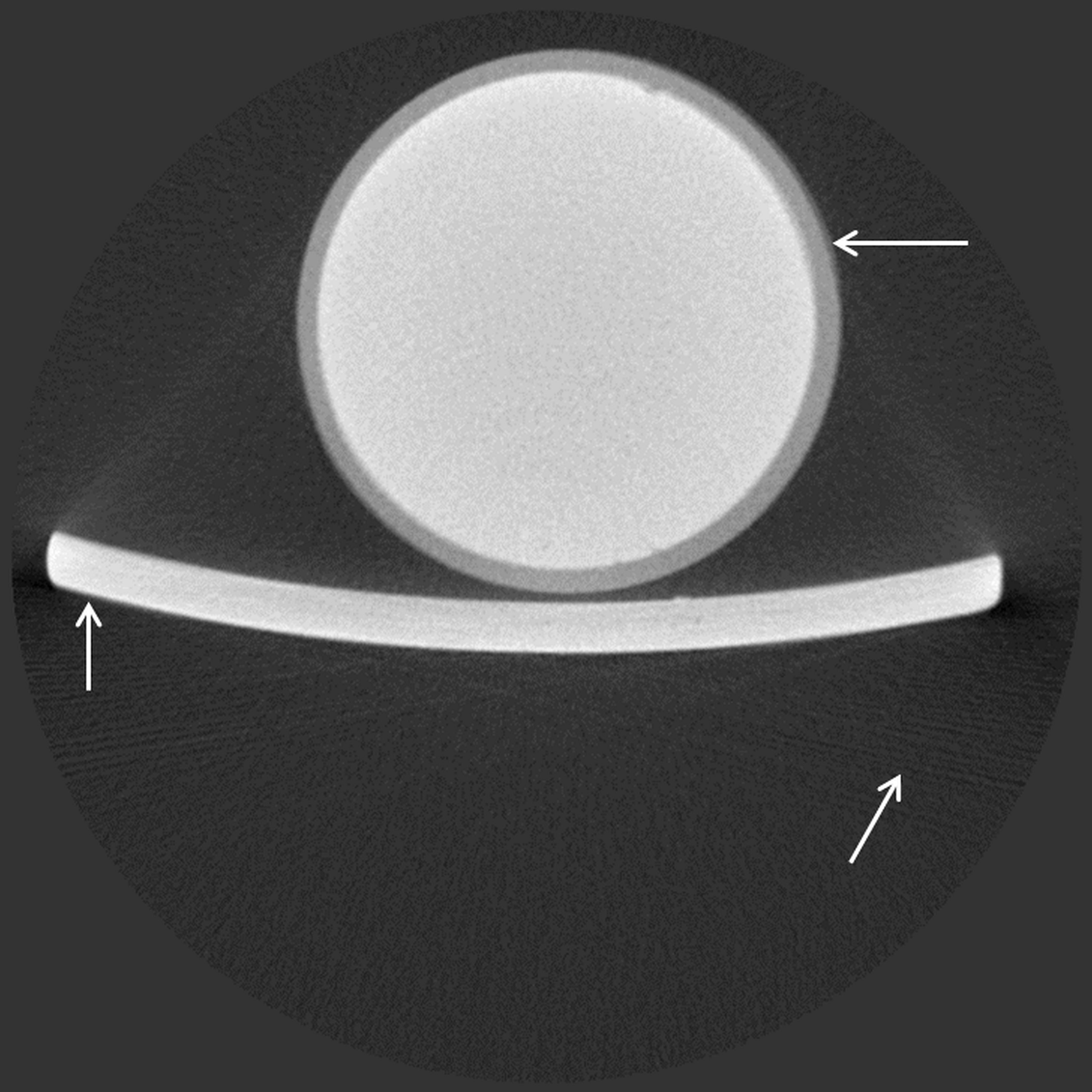

Supplement: S3 Fig — A water phantom was used to assess the sensitivity of the manufacturer’s cantilever system to machine vibrations. Motion artefacts appeared as shading or streaking in the reconstructed CT image as indicated by the arrows. (TIF) [file pone.0128537.s003.tif]

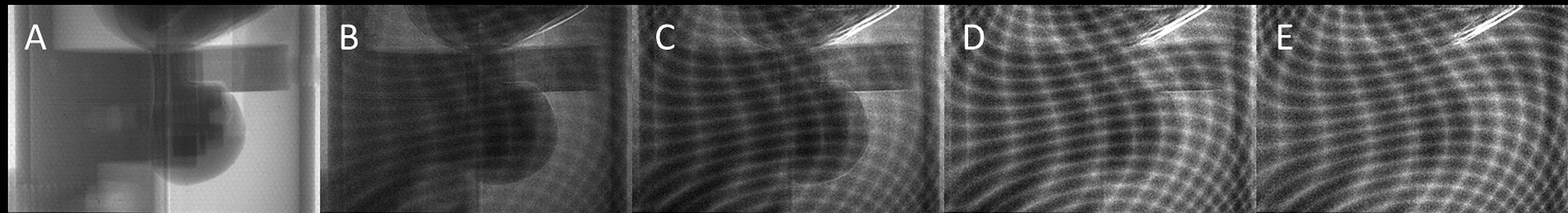

Supplement: S4 Fig — Fluoroscopy was performed during settling time of the custom cradle. Imaging was initiated immediately after mounting the cradle into the imaging system. Standard deviation of the mean is shown for 50 subsequent images. The bright edges show areas of high standard deviation and correspond to areas of large motion. A: Fluoroscopy image, B: 0–2 min, C: 5–7 min, D: 15–17 min, E: 22–24 min. The remaining wave patterns result from a temporal stability of the X-ray source. (TIF) [file pone.0128537.s004.tif]
